# Behavior Problems and Post-traumatic Stress Symptoms in Children Beginning School: A Comparison of Pre- and Post-Earthquake Groups

**DOI:** 10.1371/currents.dis.2821c82fbc27d0c2aa9e00cff532b402

**Published:** 2016-06-22

**Authors:** Kathleen Liberty, Michael Tarren-Sweeney, Sonja Macfarlane, Arindam Basu, James Reid

**Affiliations:** School of Health Sciences, University of Canterbury, Christchurch, New Zealand; School of Health Sciences, University of Canterbury, New Zealand; Centre for Brain and Mental Health Research, University of Newcastle, Australia; Te Oranga School of Human Development & Movement Studies / Te Kura Toi Tangata Faculty of Education, University of Waikato, Hamilton, New Zealand; School of Health Sciences, University of Canterbury, Christchurch, New Zealand; Deptartment of General Practice and Rural Health. Dunedin School of Medicine, University of Otago, Dunedin, New Zealand

## Abstract

**Introduction:**

Literature reviews caution that estimating the effects of disasters on the behavior of children following a disaster is difficult without baseline information and few studies report the effects of earthquakes on young children. In addition the relationship between age at the time of disaster and consequential behavior problems have not been reported for young children who experience disaster-related stress during a developmentally sensitive period.

**Methods:**

Behavior problems and symptoms of post-traumatic stress (PTS) were reported for two groups of children from nearby neighborhoods during their first term at school, using the Behavior Problem Index by teacher report, following approved informed consent procedures. Data on one group, “Pre-EQ” (N=297), was collected four years before the beginning of the earthquakes on children born 2001-2002. Data on the second group, “Post-EQ” (N=212), was collected approximately three to four years after the beginning of the earthquakes on children born 2007-2009 and living in heavily damaged neighborhoods. The Post-EQ group had significantly more children from high socioeconomic neighborhoods but no other significant differences on main demographic characteristics.

**Results:**

The mean behavior problem score was significantly higher in the Post-EQ group (Mean =6.11) as compared to the Pre-EQ group (Mean = 3.78). PTS symptoms were also significantly higher in the Post-EQ group (Mean =2.91) as compared to the Pre-EQ group (Mean=1.98) and more children had high PTS scores (20.9% v. 8.8%, OR= 2.73, 95%CI =1.57, 4.76). Model testing identified that a younger age at the time of exposure was the only significant predictor of high numbers of PTS symptoms in the Post-EQ group.

Discussion: Rates of teacher-reported behavior problems in young children more than doubled following the Christchurch earthquakes. Younger children may be more vulnerable to the effects of earthquakes that occur during a developmentally sensitive period. Additional research is needed to consider the effects of age and duration of disaster effects to better understand the effects of disasters on children, their families and communities.

## Introduction

Post-traumatic stress disorder (PTSD) is a specific psychiatric-defined mental health condition in adults and children.^1^ In children, PTSD often co-occurs with high rates of behavior problems.^1^ Risk factors for the development of PTSD following exposure to trauma include preexisting mental health and behavior problems, poverty, minority status, and gender; and, in the post-disaster environment, disruptions to normal life, perceptions of threat or reactions to specific events.^2^^,^^3^ In any community, children may develop behavior problems and PTSD associated with trauma affecting individual families (e.g., domestic violence, vehicle accidents, parent death).^2^^,^^3^ The impact of trauma interacts with risk factors and with characteristics of the particular trauma, such as its duration and intensity, as well as with family and community factors, including the availability of treatments and support.^2^^,^^3^^,^^4^ Behavior problems and post-traumatic stress (PTS) symptoms in young children are a serious mental health problem, and may be associated with continuing psychological issues, such as anxiety and conduct disorders, and may persist in adulthood.^3^^,^^4^^,^^5 ^

It has been postulated that the development of behavior problems and PTS symptoms may be influenced by age at the time of the traumatic event, particularly if it occurs during a developmentally sensitive period.^4^^,^^6^^,^^7^ Chronic traumatic stress in young children may be associated with a number of adverse biological effects, notably disruption of the limbic-hypothalamic-pituitary-adrendal axis, the hippocampus, the amygdala, the pre-frontal cortex, and other neurological structures and processes.^4^ These disruptions are likely to affect the regulation of cortisol, serotonin, dopamine and oxytocin, and are associated with emotional and behavioral problems.^4^

Unfortunately, it is difficult to determine if age at the onset of trauma is a relevant factor in the development of problems, as studies of behavior problems and PTS often group children across ages (e.g. 6-18 years) for analysis without considering developmental status or the ages of sensitive periods (e.g., 8). Although studies often report age at the time the study was conducted, they do not often report children’s age at the time of the trauma experience, or control for this in analysis.^2^^,^^9^ A meta-analysis of studies identifying risk factors for the development of PTSD in children and adolescence concluded that ‘age’ was a minor risk factor, but did not report the inclusion of any studies of young children, and did not investigate sensitive periods,^9^ possibly due to studies grouping ages across such periods in childhood and adolescence.

Young children who experience traumatic natural disasters, such as flooding, hurricanes, forest fires, earthquakes and the post-disaster period may also develop behavior problems and PTS symptoms.^2^^,^^7^^,^^10^ Exposure to earthquakes is thought to increase the risk of persistent PTS symptoms, as it is associated not only with a single traumatic event in the initial earthquake, but with continuing aftershocks, and with lengthy periods of community disruption due to infrastructure damage, damage to houses, and the disruptive nature of rebuilding.^3^^,^^11^^,^^12^ For example, 22.9% of Italian children aged 6-10 years exposed to a magnitude (M)5.7 earthquake had high numbers of PTS symptoms 12-17 months later^13^ and 28% of Turkish 8-15 year-olds had high numbers of PTS symptoms 36 months after a M7.4 earthquake.^14^

The Canterbury earthquake sequence began with a M7.1 earthquake on 4 September 2010, followed by 31 earthquakes of M>5.0 during the next four months.^15^ Then, on 22 February 2011, a shallow M6.2 earthquake with an epicenter within city limits and unprecedented upward ground acceleration velocities of 27.6 to 72.6 cm/s hit and literally lifted buildings off their foundations.^15^^,^^16^ There were 6600 injuries and 185 deaths in a population of about 440,000. Over the next eleven months, there were 32 more earthquakes of M>5.0 and approximately 14,00 aftershocks of lesser magnitude.^17^ The extended period of earthquakes over 17 months caused extensive liquefaction, destruction of 10,000-15,000 family homes, damage to more than 110,000 family homes of an estimated 140,000 homes in pre-EQ Christchurch, and a significant drop in New Zealand's gross domestic product.^16^^,^^17^^,^^18^ Christchurch experienced a significant decline of 17% in the number of households in the lowest income group following the earthquakes, which is primarily attributed to the loss of housing due to earthquake damage, particularly housing in low income neighborhoods.^18^^,^^19^

**Earthquakes in the Christchurch region, 4.10.10 to 19.09.12.**
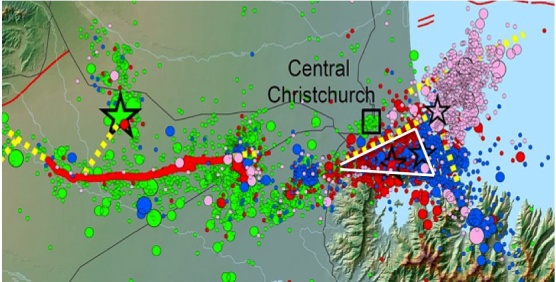
Stars indicate epicentres of major earthquakes >M6.0. The blue dots indicate aftershocks M5.0-M5.9. The dotted yellow line indicates approximate fault lines. Source: geonet. http://info.geonet.org.nz/display/home/Canterbury+Quakes ). The black square indicates the location of the central business district. The white triangle encompasses the locations of the schools and neighborhoods in this study.

Inter-spaced between earthquakes were periods of recovery and rebuilding, which would then be damaged by a subsequent aftershock. Although there were no M5.0 EQ between June 2012 and the conclusion of the study measurement period, the stressors associated with a post-disaster environment have continued, including the closure of areas to replace infrastructure, demolition of buildings and homes, loss of jobs, increased hospitalizations due to alcohol-related incidents, and so forth.^3^^,^^20^ Thirty months after the September 2010 earthquake, 78% of residents reported stress that had a 'negative impact' on their lives and 23% of the total reported that they were affected “always” or “most of the time”.^20^ Adults had a 40% increased likelihood of mental disorder two years after the February 2011 earthquake compared to those who were not earthquake-exposed.^21^ The Canterbury District Health Board reported an increase of 69% in child and youth presentations for mental health services and a 40% increase in adult referrals almost four years after the February 2011 earthquake.^22^

We sought to investigate behavior problems and PTS symptoms of children entering school who had experienced the earthquakes and subsequent post-earthquake period from early childhood through to school entry. Although children who experience earthquakes are thought to be more likely to have an increased risk of behavior problems and PTS symptoms, literature reviews do not identify studies of children exposed to earthquakes in very early childhood.^6^^,^^10^^,^^12^^,^^23^

While natural disasters may more than double baseline rates of behavior problems and PTS symptoms, very few disasters strike in areas with pre-disaster studies of behavior problems or PTS, so it is difficult to determine the effect on communities of a natural disaster.^12^^,^^23^ We were fortunate in having conducted a study of children entering school about four years before the earthquakes, and to be able to use data from that study as a baseline comparison for the present study (“Pre-EQ" group). This study involved identical measures used with children who experienced the Christchurch earthquakes and the continuing post-earthquake period up to the time of school entry, when this study was conducted (“Post-EQ" group).

The study aims were to: (1) Determine if the Post-EQ group had more children with behavior problems and PTS symptoms as compared to the Pre-EQ group at the time that the children started primary school. (2) Investigate the relationships between risk factors and the PTS symptoms in the groups, and to identify which of those risk factors independently predicted high numbers of PTS symptoms. (3) In the Post-EQ group, determine if children aged 24 months or younger (a developmentally sensitive period) at the start of the earthquake period were more likely to develop high numbers of PTS symptoms by the time they started school, as compared with children who were older at the time the earthquakes began.

## Methods

**Design**

A descriptive quantitative study was undertaken with two groups of children as they entered school, using the same measurement procedure, with the informed consent of parents, teachers and schools. Institutional ethics committees approved the recruitment and informed consent procedures for both the Pre-EQ group and the Post-EQ group. The authors affirm that the study was conducted in accordance with the World Medical Association Declaration of Helsinki.

**Participants**

*Pre-EQ Group.* The data from the Pre-EQ children are from a study of children’s health and learning at eight primary schools about 30 months prior to the start of the earthquake period.^24^ Children in this group were born between 2001 and 2002. Parents were informed about the study by the school principal or deputy principal during enrollment processes, and informed written consent for participation was gained. Children with a diagnosed disability requiring special education support, or whose first language was not English or Māori (official languages of New Zealand) were excluded. Characteristics of the group are shown in Table 1.

Principals in the Pre-EQ study schools were not able to participate in the Post-EQ research study due to school closures, mergers and other issues resulting from the earthquakes. All schools in the Pre-EQ and Post- EQ groups were located within the white triangle imposed on Figure 1. The approximate dimensions are a maximum of 12 km in the north to south axis, and 19 km in the east to west axis.

*Post-EQ Group*. The data from the Post-EQ children were collected from five different primary schools also located within the white triangle shown in Figure 1. Data were collected during the period of February 2013 through June 2014; 12 to 18 months following the M5.0 EQ in January 2012. Demographic characteristics of this group are shown in Table 1. The only significant demographic difference between the two study groups was that the Post-EQ Group had proportionally more children from high SES.

In addition to the same inclusion criteria for the Pre-EQ group, we also required that children in this group have lived in Christchurch from the first major earthquake in September 2010 through the final EQ of M.50 in January 2012, continuing through the post-disaster period to the point of measurement (the first term of primary school). Within the Post-EQ group, children’s mean age at the first large earthquake in September 2010 was 24.2 months (SD=7.4, range = 8-42).

**Figure:**
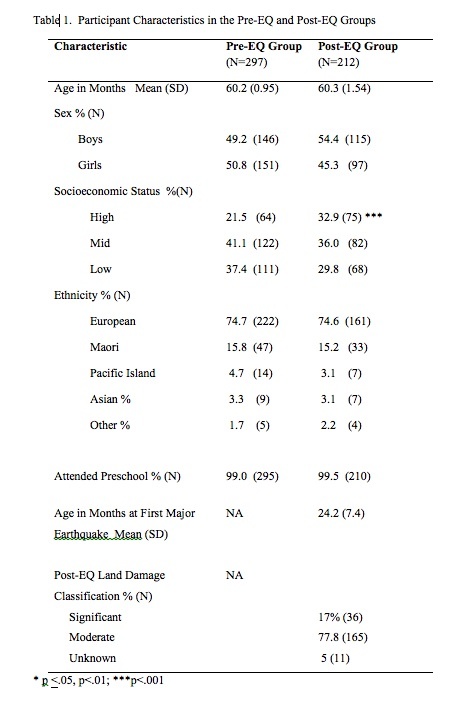

**Procedures and Measures**

Identical measures of teacher-reported behavior were used for both groups. The measurement in the Pre-EQ group occurred during 2006-2007, as they entered school at age five years. The measurement in the Post-EQ group, occurred during 2013-2014, as they entered school at age five years. For the Post-EQ group, measurement occurred a mean of 38.3 months (SD=6.3; range=29-48 months) following the first earthquake in September 2010.

*Demographic information*. With parent consent, demographic information as to gender and ethnicity was collected from school records and socioeconomic status was estimated from parent-supplied home addresses on the consent forms, using census information available to researchers.^25^^,^^26^ The government-rated earthquake damage for each Post-EQ participant's address was also recorded, as this has been shown to be significantly associated with mental health problems in families in Christchurch.^27^ All of the Post-EQ participants were living on sections of land significantly or moderately damaged by the earthquakes, with the exception of eleven participants who were living on city-owned land which does not have publicly available classification, although these addresses are on the same streets as those of participants living on moderately or significantly damaged land.

*Behavior problems*. Once informed consent was obtained from parents/caregivers, teachers completed the Behavior Problem Index^28^ based on the Child Behavior Checklist 29 which is suitable for use with five-year-old children and has been used in many studies.^30^^,^^31^^,^^32^^,^^33^ Teachers rated 26 items using a 3-point Likert scale (0=not true, 1=sometimes true, 2=often true) for a total behavior problem score. A total BPI score was calculated (range 0-52).

*PTS symptoms.* There were no resources available for individual clinical appraisal of the children, so PTS symptoms were estimated using a screening model. The most commonly used screening measures for PTS, such as the UCLA PTSD Reaction Index; Child PTSD Symptom Scale; Posttraumatic Stress Symptoms in Children, and the Trauma Symptom Checklist for Children are not suitable for children under the age of 6 years.^3^ In addition, children under the age of 6 years are not able to self-report on their own symptoms. Difficulties with measuring the symptoms of PTS in children under the age of six years are associated with their relative developmental immaturity, their limited communicative competence in understanding their own emotions, the lack of development of executive function needed to differentiate thoughts from emotions and memories from present events, and so forth.^5^^,^^10^ This difficulty has been recognized in continuing examination and consideration of how PTS symptoms are expressed in the behavior of young children, and as represented in the introduction of new diagnostic criteria for PTSD in children, with a separate set of symptom criteria for children aged six and younger.^1^

In order to provide a screening estimation of PTS symptoms, the present study used dichotomized scores for symptoms identified by Dehon and Scheeringa 34 as represented in items from the BPI (Items can be seen in Table 2). Dehon and Scheeringa 34 also used dichotomized scoring in their preschool scale, because the weight of symptom frequency has not been sufficiently investigated. Therefore, we applied dichotomous scoring, with items marked by teachers as ‘sometimes true'; and ‘often true’ scored as “1” and items marked ‘not true’ scored as 0, yielding a possible score range of 0-10.^34^^,^^35^

**Statistical analysis**

In the first step of the analysis, we compared the mean total BPI score between the groups, the total PTS score, and an adjusted BPI score after removing the PTS items, using t-tests with 95% as a level of confidence. The next step was to examine the presence of high symptoms in both groups, using odds ratios. We used the 95% percentile in the Pre-EQ group as the cut-point for high symptom levels. The Pre-EQ group was the reference group for the odds ratios (OR) calculations. The third step was to analyze the behavior problem and PTS sub-scores, as well as the demographic characteristics of children who were hypothesized to be in different sensitive periods at the start of the earthquake exposure period based on their chronological age. Finally, logistic regression was used to test predictive models for high PTS symptoms (high symptom score vs. all other scores). Demographic variables were dichotomized by risk status identified in previous studies (i.e., gender was dichotomized as girl v. boy, SES was dichotomized as low SES v. all else; ethnicity was dichotomized as European v. minority status). The model testing for the Post-EQ group included the addition of two additional variables in Steps 2 and 3. In Step 2, land damage at the location of the child’s house was dichotomized as severe v. moderate.^25^ In Step 3, children’s age at the start of the earthquake period was dichotomized as < 24 months (N=110) or >24 months (N=112). From birth to 24 months of age has been identified as a sensitive period for initial development of expressive and receptive language, confident independent mobility, and competent imitation of a model, and has been associated with sensitive periods in brain development.^4^^,^^6^^,^^39^ All analyses were performed using the Statistical Program for Social Sciences version 21.

## Results

Children in the Post-EQ group had significantly higher scores for total behavior problem scores and post-traumatic symptoms scores (Table 2). In addition, children in the Post-EQ group had significantly more PTS symptoms (Table 2). A score of >6 (i.e., >60% of the 10 items we identified) was used to categorize high symptom scores. Dehon and Scheeringa^34^ identified that children scoring 60% of symptom items was a useful screening marker, which was the equivalent of our cut-off for a high screening score.

**Figure:**
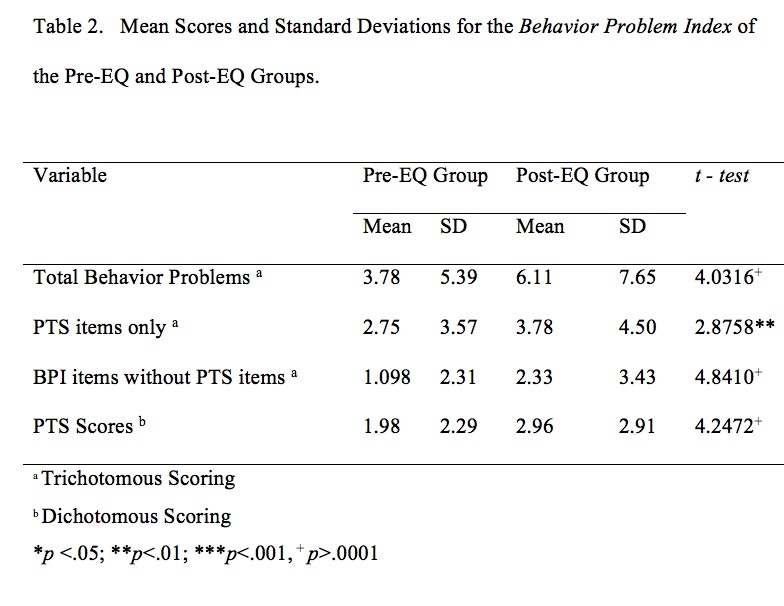

**Figure:**
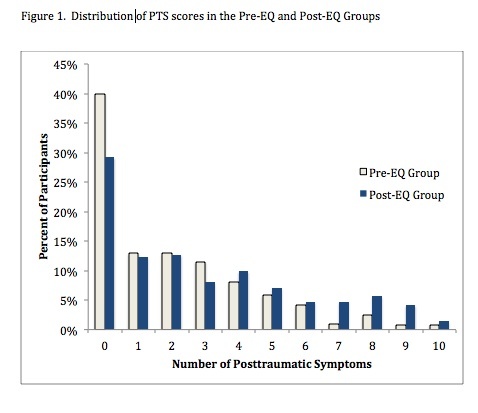

Significantly more children in the Post-EQ group (20.7%) had high PTS symptom scores as compared to the Pre-EQ group (8.8%) (Table 3). Children in the Post-EQ group were also more likely to demonstrate 8 of 10 individual PTS symptoms as compared to the Pre-EQ group (Table 3). The Post-EQ group showed increased Odds Ratio for high numbers of PTS symptoms for boys, girls, European ethnicity, and both low and high SES neighborhoods (Table 4), but not for Māori children.

**Figure:**
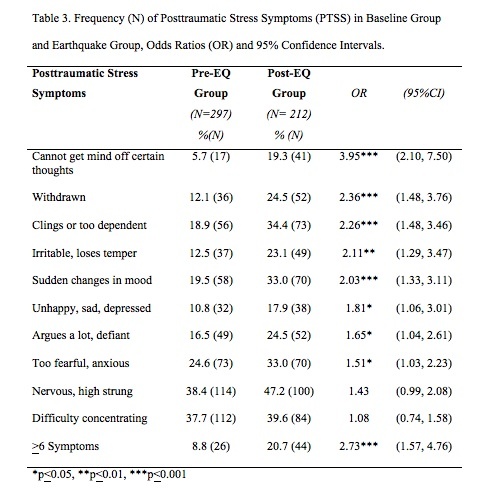

**Figure:**
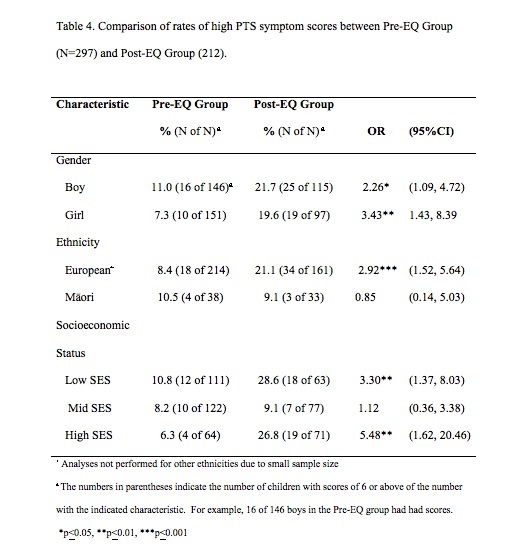

Scores of children in the Post-EQ group were sub-divided into two groups, based on the child's chronological age at the start of the earthquake period. Data from children aged 24 months and younger (range 8-24 months) comprised the “Younger” group (N=102), and data from the other 110 children were categorized as “Older” (range 25-42 months). Comparisons identified that the groups were significantly different in age, and in the length of time from the start of the earthquake period to the time of measurement – the duration of their exposure to the earthquakes and the post-earthquake stressors (Table 5). Most importantly, the younger group was significantly more likely to have higher behavior problem and PTS scores. There were 29.1% of children in the “younger” group with PTS scores of 6 or more as compared to 11.8% of children in the “older” group (OR=3.08, 95%CI= 1.41, 6.82), and the only significant demographic difference between these groups was that more of the younger group were living on significantly damaged land.

**Figure:**
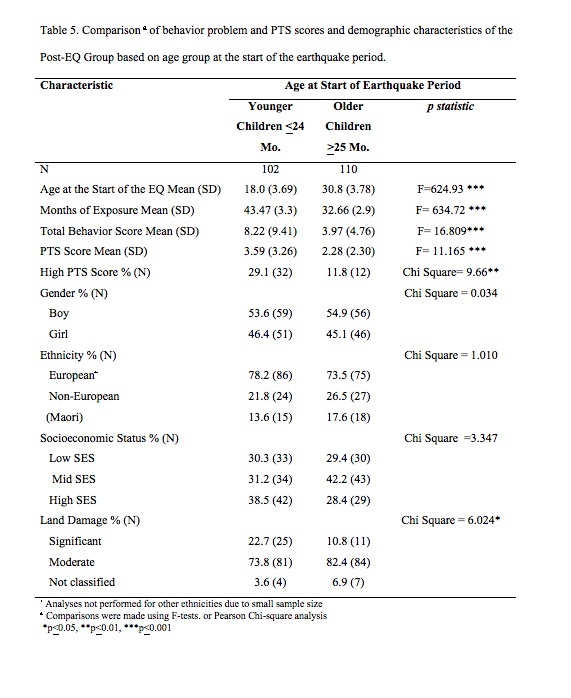

Logistic regression models were performed to identify independent predictors of high numbers of PTS symptoms (Table 6). Step 1 testing identified that there were no independent predictors of high PTS symptom score in either the Pre-EQ or the Post-EQ groups, and none of the variables tested reached significance.

We then tested two additional variables related to the Post-EQ group. In Step 2, the severity of land damage did not improve the model. For Step 3, we added a dichotomized variable of age at the start of the earthquake period. When the younger v. older age at the start of the earthquakes factor was added to the model testing in Step 3 (Table 6), the model reached significance (X^_2_^=15.217, p=.009) and younger age at the start of the exposure period was the only independent predictor identified.

**Figure:**
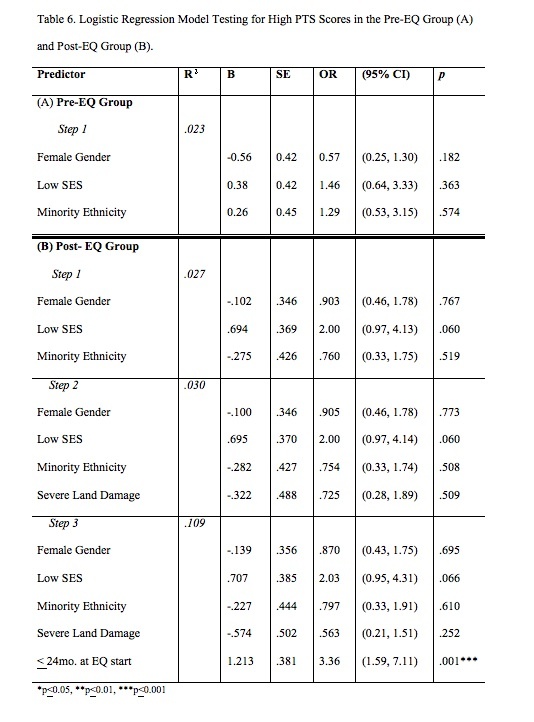

## Discussion and Conclusions

This is the first study to report behavior problems and PTS symptoms among children who had lengthy experience of earthquakes and post-disaster stressors during early childhood as compared to a demographically similar group from similar and nearby neighborhoods who did not experience the disaster. The major finding showed that there were significantly more behaviour problems and post-traumatic stress symptoms in the children who entered school after the earthquakes. The rate of PTS symptoms in the Pre-EQ Group is comparable to rates in non-disaster exposed communities.^2^^,^^4^ The rate of children with high PTS scores in the Post-EQ group is similar to that reported for older children who experienced earthquakes or other disasters,however, there is no comparable study of young children.^3^^,^^8^^,^^9^^,^^13^^,^^14^^,^^36^^,^^37^ The reports by the local health authority of a post-EQ increase of 69% in child mental health referrals 22 supports our results.

Many of the demographic factors associated with the development of PTS symptoms were associated with higher PTS scores in the Post-EQ group as compared to the Pre-EQ group. These relationships were expected for the demographic variables tested and are similar to other studies.^23^ However, there were two important discrepancies. One discrepancy was that EQ children in the mid-SES group did not show significantly increased rates of high PTS scores over the Pre-EQ group, while children in the low-SES and high SES groups both showed a significant increase. Although these differences may be due to small sample sizes, high- and mid- SES are not necessarily a protective factor for post-disaster PTS 3^,^^37^ and in the present study, SES was not a significant predictor for high PTS scores for either the Pre-EQ group or the Post-EQ group. A similar result was also reported in a study involving adults who experienced the Christchurch earthquakes.^27^ This suggests that other factors, such as the age at the start of the exposure period, are more likely to be related to the overall increase in high PTS levels than SES. This is one aspect that should be further examined.

The second difference was for Māori children, who did not show a significant increase in rates of high PTS symptoms in the Post-EQ group. This may also be due to low numbers of children in the study participants. However, research indicates that the social connectedness, spiritual support and collective dynamics that are associated within Māori communities in Christchurch may have protected them against the development of high PTS symptoms following the earthquakes 21^,^^27^^,^^38^ Hogg and colleagues^27^ also reported that Māori adults in highly earthquake-affected Christchurch neighborhoods were less likely to have a mood or anxiety disorder as compared to other ethnic groups. The rates in Māori study children may therefore be reflective of fewer mental health problems in their parents or community.

The only independent predictor of high PTS scores identified in the regression testing was ‘age at the beginning of the earthquake period’. In the present study, children who were aged 24 months or younger at the time of the first major earthquake had almost triple the rates of high PTS as compared with the baseline Pre-EQ group. In addition, their rates were higher than the "older group", who had spent the same amount of time in the earthquake period, but were older at the start of the exposure period. Thus, this younger Post-EQ group had experienced a longer time period potentially marked by traumatic events that might have affected their emotional and behavioral development, as compared to children who were older at the time of the first earthquake. Although this is a small sample size, the data support the findings of other researchers who have suggested that traumatic stress at key developmental stages in early childhood can have long-lasting negative effects.^6^^,^^7^^,^^39^^,^^40^

Our findings are limited in a number of ways. First, the study is confined by problems common to post-disaster research– small sample size and lack of randomization.^9^^,^^36^ In addition, our findings may be limited by the fact that teachers had only known the children for a relatively short period of time before reporting on their symptoms, but this was identical for both the baseline and Post-EQ groups. Also, our study had fewer children from low SES and more children from high SES as compared to the Pre-EQ group, as the city experienced a drop in low income families due to the destruction of housing in the poor neighborhoods in the neighborhoods of participating schools. As low SES children are more vulnerable to developing PTS symptom,^3^ our relatively low rate of children from low SES limits the external validity of our results and indicates that our results may underestimate the rate of behavior problems and PTS symptoms.

Second, there are important limitations to our measurement. There was no clinical examination of the children by trained psychiatrists or clinicians, and the data represent the results of a behavior screening scale completed by teachers. This is an important limitation because teachers are not trained in diagnosis of behavior problems, and thus teacher reports may not represent clinical diagnosis. However, many studies and clinical diagnostic protocols use parent reports.^1^^,^^3^^,^^5^^,^^37^ Compared to parents, teachers may report more conservatively on children’s symptoms, possibly due to the fact that they have a far wider experience base of typical and atypical behaviors, different expectations, or perceptions as compared to parents.^41^ For example, the PTS of 138 three to five year old children exposed to a traumatic event were rated by parents and teachers: significantly, parents rated irritability in 29% of the children as compared to 7.1% rated by teachers; similarly, parents rated clingy behavior in 46.7% as compared to 7.2% by teachers.^42^ Also, there is only a single informant for behavior problems and PTS symptoms, and, although this is also similar to 70% of disaster studies,^43^ it is possible that behavior problems and PTS has been under-estimated or over-estimated or otherwise limited in terms of accuracy. Finally, we did not have a measure of the child’s mental health or developmental status at the start of the earthquakes, which are both risk factors for the development of behavior problems^2^^,^^6^^,^^9^^,^^39^^,^^43^ thus some children may have shown problems prior to the earthquakes. However, the baseline rates we have available for comparison, and the literature on PTS in children following earthquakes, indicate that it is more likely that increased behavior problems and PTS are associated with disaster exposure, but other possibilities can not be ruled out.

Despite these limitations, the study findings make an important contribution to our understanding of the complex effects of an extended duration of earthquakes and post-disaster stressors experienced during early childhood on child mental health. Our study findings demonstrate that pre-disaster rates of behavior problems and PTS symptoms in children entering primary school more than doubled following their experiences of growing up during earthquakes and post-earthquake stressors, similar to the results of other studies. These changes will have implications for families, schools, and mental health service providers, as well as community disaster planning.

Children who were two years of age or younger at the start of the earthquakes were far more likely to have negative impacts on their mental health. Age at the start of traumatic events, particularly chronic or long-lasting events, may have important implications for treatment. Future studies should report children’s age at the start of any disaster period, and avoid combining results across age groups and sensitive developmental periods during analysis. This is particularly important as natural disasters associated with climate change are predicted to increase, with concomitant increased risk for poor mental health.^44^^,^^45^ Studies that report and consider chronological age at the time of trauma exposure would allow us to better understand the complex effects of natural disasters on children’s mental health and to plan and consider developmentally appropriate therapies. Similarly, studies of children who experience potentially traumatic events such as war, terrorism or migration^4^^,^^43^^,^^46^ should investigate the relationships between children’s age at the beginning of the trauma period and their mental health outcomes. The vulnerability of young children to traumatic events and their subsequent mental health requires a sustained research focus.

## Competing Interests

This research was funded in part by the Asthma and Respiratory Foundation of New Zealand (Pre-EQ group) and the internal grants programme of the University of Canterbury (Post-EQ group). Neither of the funders played any role in the design, execution, interpretation or dissemination of the results of this study. None of the authors have competing interests in regards to this research study.
